# Exploratory Analysis of Regulated Cell Death-Related Genes as Potential Prognostic Biomarkers in Endometrial Carcinoma

**DOI:** 10.3390/biomedicines13092289

**Published:** 2025-09-17

**Authors:** Yu-Xuan Lin, Dong-Yan Cao

**Affiliations:** National Clinical Research Center for Obstetric & Gynecologic Diseases, Department of Obstetrics and Gynecology, Peking Union Medical College Hospital, Chinese Academy of Medical Sciences & Peking Union Medical College, Peking Union Medical College Hospital (Dongdan Campus), Beijing 100730, China

**Keywords:** endometrial carcinoma, regulated cell death, TCGA, GEO

## Abstract

**Objective:** This study aims to explore the mechanism of regulated cell death-related genes in the development of endometrial carcinoma. **Methods:** Endometrial carcinoma-related datasets were yielded via the Cancer Genome Atlas and Gene Expression Omnibus databases, and regulated cell death-related genes were extracted from the literature. Differential expression analysis, weighted gene co-expression network analysis, and protein interaction analysis were performed to identify critical regulated cell death-related genes. Gene set enrichment analysis was used to identify the functional pathways involved in these critical genes. Afterward, the best clustering approach for tumor samples was yielded via consensus clustering analysis, and nomogram prediction models were built. Shiny Methylation Analysis Resource Tool was used to compare the expression levels of CpG methylation probes for critical genes between tumor and normal samples. Spearman correlation analysis was conducted to investigate the relationship between critical genes and various immune features. Eventually, immuno-infiltrative analysis was implemented, and potential therapeutic agents were screened targeting critical genes. The data were analyzed and visualized by R software using different packages. In addition, the expressions of critical genes were validated by quantitative real-time polymerase chain reaction and immunochemistry. **Results:** Four critical genes, namely *GBP2*, *SLC11A1*, *P2RX7*, and *HCLS1*, were identified, and they were involved in various functional pathways such as leukocyte-mediated cytotoxicity. There were substantial differences in CpG methylation in *GBP2*, *SLC11A1*, and *HCLS1* between tumor and normal samples. As for immune features, all critical genes were positively connected with immunosuppressive factors such as TIGIT and most HLA molecules in endometrial carcinoma. The critical genes high/low expression groups of tumor samples showed different immune responses towards PD-1, PD-L1, and CTLA-4 immunotherapy. The infiltration of 24 immune cells, such as effector memory CD8 T cells, was notably different between tumor and normal samples. Based on sensitivity analysis of chemotherapeutic agents, we found the highest positive correlation between *SLC11A1* and “BI.2536” and the strongest passive correlation of *HCLS1* and *GBP2* with “Ribociclib”, as well as *P2RX7* with “BMS.754807”. Quantitative real-time polymerase chain reaction suggested that the expression trends of *GBP2*, *P2RX7*, and *HCLS1* were consistent with the results of bioinformatic analysis. **Conclusions:** Regulated cell death-related genes (*GBP2*, *SLC11A1*, *P2RX7*, and *HCLS1*) may play a role in endometrial carcinoma development, which can provide new ideas for the treatment and prognosis prediction of this disease.

## 1. Introduction

Endometrial carcinoma (EC) is the most common gynecological malignancy globally [1], and its incidence is still on the rise [1]. Although the five-year survival rate for patients with early EC appears promising, the prognoses for recurrent and advanced EC are both dismal [1]. Chemotherapy is regarded as the foundation of adjuvant therapy for patients with extrauterine disease, but the response rate is only about 40% to 62%, and overall survival ranges from 13 to 29 months [2]. The rapid development of tumor molecular biology has deepened our understanding that EC is not only a genetic disease but also an immune disease [3]. Chemotherapy combined with immunotherapy has emerged as a breakthrough treatment for patients with mismatch repair deficient tumors, as the survival outcomes of this combined regimen (such as the addition of programmed death protein-1/programmed cell death ligand-1 [PD-1/PD-L1] blockade to chemotherapy) have been shown to be better than chemotherapy alone in many randomized prospective studies [4,5]. Existing biomarkers that help predict immunotherapy response include target antigen expression, tumor mutational burden, DNA mismatch repair deficiency, and tumors at baseline T cell infiltration [4,5]. However, there are still lots of potential responders who are excluded from first-line immunotherapy due to a lack of alternative predictive biomarkers and new targets. Generally, challenges remain, and there is still a need for new biological markers to aid in definitive diagnosis of EC as well as new targets for developing more effective treatments.

Regulated cell death (RCD) refers to the active and orderly death of cells generated by gene regulation to maintain homeostasis in organisms under physiological and pathological conditions [6,7]. The induction and execution of RCD are mainly regulated by the formation of signal amplification complexes, which also play an essential role in immune response [6,7]. Currently known RCD types include autophagy-dependent cell death, apoptosis, netotic cell death, pyroptosis, ferroptosis, parthanatos, entosis, necroptosis, and so on [7]. Each type of RCD shows immunomodulatory characteristics, from anti-inflammatory and tolerance to promoting inflammation and immunogenicity [7]. Avoiding RCD is an important sign of cancer, and tumor cells may have the characteristic of anti-tumor treatment because of the mutation that destroys subroutines of RCD [8,9]. Therefore, RCD can affect tumor development and treatment outcome by regulating progression, immune microenvironment, chemoresistance, and so on [8,9]. Targeted therapy combined with immunotherapy against the above RCD pathways may exert powerful antitumor activity [10], which also provides a new direction of treatment of patients with recurrent or advanced EC in the future. Recent studies have already revealed the interaction between RCD and antitumor immunity in liver cancer, lung cancer, gastrointestinal cancer, and so on [8,9,10]. However, related mechanisms may differ in the EC and have not been fully disclosed till now.

In this study, we obtained RCD-related genes (RCD-RGs) based on various public datasets. Comprehensive bioinformatics analyses were then carried out on these genes, and a nomogram prediction model was built. Then we further investigated infiltration features, immunotherapy responses, and potential therapeutic agents targeting critical genes. The findings may provide new ideas for the diagnosis of EC and the development of new drugs and treatments.

## 2. Materials and Methods

### 2.1. Sources of Data

Based on the sample size, quality, completeness of clinical information of the datasets, and their matching with the research objectives, this study selected The Cancer Genome Atlas (TCGA)-EC dataset (548 EC samples and 35 normal samples) and the GSE17025 validation set (91 EC samples and 12 normal samples, with the test platform being GPL570 [HG-U133 Plus 2] Affymetrix Human Genome U133 Plus 2.0 Array), which were obtained from the TCGA (https://portal.gdc.cancer.gov/ [accessed on 3 April 2023]) and Gene Expression Omnibus (GEO) (https://www.ncbi.nlm.nih.gov/gds [accessed on 3 April 2023]) databases, respectively. Both datasets were mRNA transcriptome sequencing data. In the TCGA-EC dataset, 544 EC samples had complete survival information. The clinical characteristics of samples were shown in Table S1. We extracted 4392 RCD-RGs (3259 were yielded after the de-duplication) from the literature, as this article had already selected all the known RCD-RGs from the FerrDb database (http://www.zhounan.org/ferrdb [accessed on 3 April 2023]), the HADb database (http://www.autophagy.lu/ [accessed on 3 April 2023]), and several published studies [7]. These RCD-RGs are well-recognized genes with established associations with regulated cell death processes, independent of the candidate genes ultimately identified in this study.

Subsequently, the 548 EC samples in the TCGA-EC dataset were subclassified into 405 cases of “Adenomas and Adenocarcinomas”, 141 cases of “Cystic, Mucinous and Serous Neoplasms”, and 2 cases of “Epithelial Neoplasms, Not Otherwise Specified (NOS)”. Due to the small sample size of the “Epithelial Neoplasms, NOS” subtype, 405 cases of ‘Adenomas and Adenocarcinomas” and 141 cases of “Cystic, Mucinous and Serous Neoplasms” were selected for further analysis.

### 2.2. Differential Expression Analysis and Weighted Gene Co-Expression Network Analysis

In the TCGA-EC dataset, differentially expressed genes (DEGs) between tumor and normal samples were sifted out by the DESeq2 package (version 1.36.0) [11], setting adj. *p* < 0.05 and |log_2_FC| > 1. To quantify the overall activity of RCD pathways in each sample, we calculated gene set variation analysis (GSVA) scores based on the expression profiles of these 3259 predefined RCD-RGs. This scoring system reflects the global activation or suppression status of RCD pathways in the samples. Therefore, based on the RCD-RGs, we calculated GSVA scores for EC samples, which were considered as traits for weighted gene co-expression network analysis (WGCNA). First, samples were clustered to remove outliers, and a soft threshold was determined for the data (R^2^ = 0.9). Then, we yielded gene modules by constructing co-expression matrix (MEDissThres = 0.25). Furthermore, the correlation between gene modules and GSVA score was analyzed, and the module with the highest correlation with GSVA score was selected as the key module (|correlation(cor)| > 0.3, *p* < 0.05).

### 2.3. Acquisition of Critical Genes

To ensure that intersected genes not only showed differential expression but also were associated with RCD functions, the intersection of DEGs and key module genes obtained from WGCNA was used to identify critical genes, thereby achieving associations with both phenotypic differences and functional pathways. Then they were analyzed for gene ontology (GO) and Kyoto Encyclopedia of Genes and Genomes (KEGG) functional enrichment by setting adj. *p* < 0.05. To explore whether there were interaction relationships among intersected genes, we constructed a protein–protein interaction (PPI) network (removed the discrete proteins) via the STRING database (http://string-db.org [accessed on 3 April 2023]). After that, intersected genes were scored utilizing ten algorithms (MCC, DMNC, MNC, Degree, EPC, BottleNeck, EcCentricity, Closeness, Radiality, and Betweenness) in Cytohubba software 3.10.1, and the top 40 genes gained by each algorithm were taken in intersection to identify the critical genes. In the TCGA-EC dataset, we implemented receiver operating characteristic (ROC) analysis on critical genes utilizing the pROC package (version 1.18.0) [12] to explore their predictive power for EC patients, and we also compared the differences in expression of critical genes between EC and normal samples by Wilcox test (*p* < 0.05). Moreover, the assessment results and differences in expression of critical genes were validated in the GSE17025 validation set by the same methods. To explore the functional pathways involved in the critical genes in the TCGA-ECdataset, we applied single gene set enrichment analysis (GSEA) by clusterProfiler package (version 3.8.1) [13], setting adj. *p* < 0.05. Finally, in the context of the TCGA-EC dataset, Pearson correlation coefficients between the four core genes were calculated using the rcorr() function from the PerformanceAnalytics package (Version 2.0.8), with the statistical thresholds set as an absolute correlation coefficient |cor| > 0.3 and *p* < 0.05.

### 2.4. Differential Expression of Critical Genes in Subgroups

Based on the different subtypes of EC in the TCGA-EC dataset, the Wilcoxon test was used to analyze the differences in the expression of key genes between the two subtypes and normal samples (*p* < 0.05). The analysis results were visualized using the ggplot2 package (version 3.5.2, https://ggplot2.tidyverse.org [accessed on 4 April 2023]).

### 2.5. Consensus Clustering Analysis

Based on the critical genes, we implemented consensus clustering analysis on the EC samples in the TCGA-EC dataset via the ConsensusClusterPlus package (version 1.60.0) [14] (clusterAlg = “pam”, distance = “canberra”), and the best clustering form was yielded according to the cumulative distribution function (CDF) value. Subsequently, we implemented the principal component analysis (PCA), t-distributed stochastic neighbor embedding (t-SNE), and survival analyses on the clusters. In addition, we also analyzed the clinicopathological characteristics (G stage, tumor stage, age, body mass index, radiation therapy, and pregnancies) between clusters.

### 2.6. Evaluation of Critical Genes

The optimal threshold of critical genes was gained via the “surv cutpoint” function, and EC samples (544 samples had complete survival information) were divided into critical gene high/low expression groups for survival analysis based on the optimal threshold (log-rank test, *p* < 0.05). To further realize the clinical value of the critical genes, we constructed a nomogram based on the expression of critical genes by the rms package (version 5.1-4) [15]. Each gene in the nomogram corresponds to a score, and the scores for all genes are summed to the total score. The total score of the sample is used to predict the probability of EC, with higher scores representing a higher prevalence. Then the calibration curve and ROC curve (AUC > 0.7) were plotted to assess the validity of the nomogram. Moreover, the decision curve analysis (DCA) curve was drawn to evaluate the degree of patients’ benefit. Meanwhile, to investigate whether the key genes possess specific diagnostic value, nomograms were constructed and evaluated using the same method in the “Adenomas and Adenocarcinomas” and “Cystic, Mucinous and Serous Neoplasms” subtypes, respectively.

### 2.7. Analysis of CpG Methylation and Response to Immunotherapy

The expression levels of CpG methylation probes for critical genes were compared between tumors and controls using the Shiny Methylation Analysis Resource Tool (SMART, http://www.bioinfo-zs.com/smartapp [accessed on 30 January 2024]) (*p* < 0.05). Meanwhile, to investigate the relationship between critical genes and various immune features, Spearman correlation analysis of critical genes with immune activators, major histocompatibility complex (MHC), and immunosuppressants was carried out in TCGA-EC using the TISIDB (http://cis.hku.hk/TISIDB/index.php [accessed on 30 January 2024]) database. Additionally, the Cancer Immunome Atlas (TCIA, https://tcia.at/ [accessed on 30 January 2024]) evaluated the immunophenotypic scores (IPS) of EC patients (including anti-PD-1/PD-L1 treatment and anti-CTLA-4) and conducted a *p* < 0.05 difference analysis across groups.

### 2.8. Immuno-Infiltrative Analysis

To further understand the role of critical genes in immune infiltration, our study evaluated the immune infiltrating cell types in all samples from the TCGA-EC dataset via the single-sample gene set enrichment analysis (ssGSEA) algorithm. First, the difference in immune cell infiltration between tumor and normal samples was analyzed utilizing the Wilcox test (*p* < 0.05), and we also analyzed the Spearman correlations between critical genes and immune cells and among immune cells, respectively, (|cor| > 0.3, *p* < 0.05). Eventually, 18 immune checkpoint molecules were selected from the literature [16] (Table S2), and the Spearman correlation between critical genes and immune checkpoint molecules was analyzed (|cor| > 0.3, *p* < 0.05). Furthermore, to clarify the immune infiltration characteristics of different subtypes, we also conducted immune infiltration analysis in the “Adenomas and Adenocarcinomas” and “Cystic, Mucinous and Serous Neoplasms” subtypes.

### 2.9. Prediction of Potential Therapeutic Agents

In the TCGA-EC dataset, we utilized the oncoPredict algorithm to predict the 50% inhibitory concentration (IC_50_) of 198 drugs via the Genomics of Drug Sensitivity in Cancer (GDSC) database (https://www.cancerrxgene.org/ [accessed on 9 May 2023]), and they were subjected to Spearman correlation analysis with critical genes (|cor| > 0.3, *p* < 0.05). The drugs with the highest correlation with critical genes were extracted, and the IC_50_ of them was compared between critical gene high/low expression groups (*p* < 0.05).

### 2.10. Immunohistochemistry (IHC)

The tissues of EC (number of samples = 10) and normal (number of samples = 10) groups were collected from Peking Union Medical College Hospital. Two pathologists were invited to examine all the samples. Only the samples that were free of potential lesions (including simple hyperplasia, complex hyperplasia, atypical hyperplasia, and so on) could be included in the normal group. First, specimens were fixed with 4% paraformaldehyde at room temperature for 24 to 48 h. Sections of these fixed specimens were then cut into 3 μm thick paraffin blocks. *HCLS1* antibody (1:100; absin; abs130408), *GBP2* antibody (1:100; absin; abs132878), *P2RX7* antibody (1:50; absin; abs137612), and *SLC11A1* antibody (1:200; abcam; ab124802) were incubated overnight at 4 °C, and anti-rabbit immunoglobulin G (1:200; abcam; ab205718) was incubated at 37 °C for 20 min. Finally, the sections were visualized under a microscope or scanned by the SQS-12P Slide Scanner (Shengqiang, Shenzhen, China). After scanning, the results were analyzed using ImageJ-pro-plus software, and GraphPad Prism 10.1.2 was used to generate bar graphs to statistically analyze the differences in positive rates (%) of critical genes between the tumor group and the normal group (*p* < 0.05). The criteria for determining positivity were as follows: cytoplasmic, membrane, or nuclear staining with brownish-yellow granular deposits was considered positive (adjusted according to the known localization of each protein). No staining or staining intensity consistent with the background was regarded as negative. In addition, the positive rate (%) was defined as the percentage of the positive area relative to the total area, which was measured using ImageJ-pro-plus software. For each section, 5 non-overlapping fields of view were randomly selected under a 200× magnification. The ratio of the brownish-yellow positive stained area to the total tissue area in each field of view was calculated, and the average value was taken as the final positive rate of the sample. The evaluation adopted a semi-quantitative scoring system combined with a counting method [17].

### 2.11. The qRT-PCR

The frozen tissues of EC (number of samples = 10) and normal (number of samples = 10) groups were also collected through the same process mentioned above. The 50 mg tissues were separately taken from each sample, and total RNA was extracted with TRIzol reagent. Next, we took 1 μL of RNA and detected the concentration of them with NanoPhotometer N50 (Implen, Munich, Germany). The mRNA was reverse transcribed using the surescript-first-strand-cDNA synthesis kit from Servicebio. The reverse transcription product cDNA was diluted 5–20 times with ddH_2_O (RNase/DNase free), and then the quantitative real-time polymerase chain reaction was subjected. The internal reference for gene detection was Glyceraldehyde-3-Phosphate Dehydrogenase (GAPDH). Finally, the expressions of critical genes between tumor and normal groups were compared (*p* < 0.05). Primer sequences were shown in Table S3.

### 2.12. Statistical Analysis

The data of IHC and qRT-PCR were analyzed and visualized by Graphpad prism software 8.0.1.244. The rest of the data were analyzed and visualized by R software 4.2.2 using different packages. *p* < 0.05 indicates that the difference is statistically significant.

## 3. Results

### 3.1. Identification of Differentially Expressed Genes and Key Module

**Figure 1:**
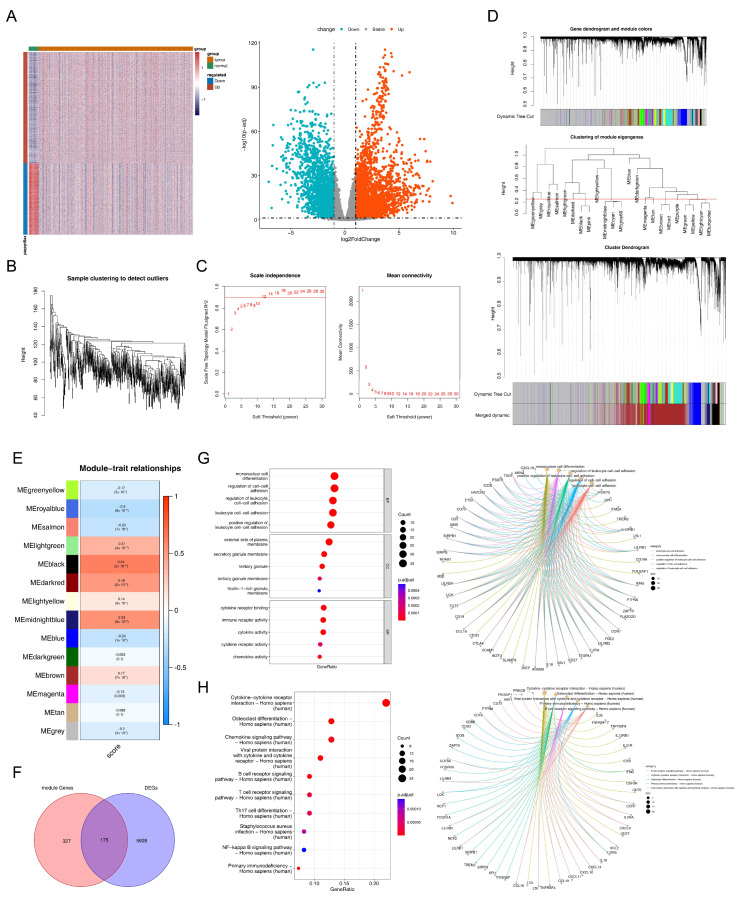
Differentially expressed genes and intersected genes. (**A**) Heatmap (left) and volcano plot (right) of differentially expressed genes (DEGs). In the heatmap, red indicates high expression and blue indicates low expression. (**B**) Sample clustering of the dataset samples. The x-axis represents samples, and the y-axis, “Height”, represents the differential distance between samples (reflecting the dissimilarity of expression patterns/data characteristics). (**C**) Determination of the power threshold. Left panel: Relationship between soft threshold (power) and scale-free network fitness (Scale Free Topology Model Fit, R^2^). The numbers represent different R^2^ values. Right panel: Relationship between soft threshold and mean connectivity. The numbers represent different soft thresholds. (**D**) Construction of co-expression matrix. Top left: Dynamic cutting tree of modules. Top right: Clustering tree for merging similar modules. Bottom left: Dynamic cutting tree for merging similar modules. Different colors represent different clustering modules. (**E**) Gene set variation analysis score correlation heat map. Left panel: Co-expression modules identified by weighted gene co-expression network analysis (WGCNA). Right panel: The color and numerical values of the color blocks represent the correlation coefficients between modules and phenotypic traits (score) (*p* values are in parentheses). Red indicates a positive correlation, and blue indicates a negative correlation. (**F**) Venn diagram of module genes and differentially expressed genes. (**G**) Gene ontology (GO) enrichment analysis of intersected genes. Left panel: GO bubble plot. The y-axis represents enriched GO terms, and the x-axis, “GeneRatio”, refers to the proportion of enriched genes in the target gene set. The size of the bubbles corresponds to “Count” (number of enriched genes), and the color represents “*p*.adjust” (adjusted *p*-value; smaller values indicate higher significance). Right panel: Gene-GO term network diagram, where gray dots represent genes and yellow dots represent GO terms. (**H**) Kyoto Encyclopedia of Genes and Genomes (KEGG) enrichment analysis of intersected genes. Left panel: KEGG bubble plot. Right panel: Gene-KEGG pathway network diagram.

There were 6103 DEGs between tumor and normal samples in the TCGA-EC dataset, including 3720 with up-regulation and 2383 with down-regulation (Figure 1A, Table S4). With respect to WGCNA, we discovered that the overall clustering of the samples in the TCGA-EC dataset was united (Figure 1B); thus, there was no need to eliminate outlier samples. In addition, when the power threshold was determined to be 12, the network approached a scale-free distribution (R^2^ = 0.9) (Figure 1C). At this point, the vertical coordinate R^2^ exceeded 0.9, and the mean value of the adjacency function was gradually closing to 0, indicating that the network approached the scale-free distribution and showed a flat trend. Afterward, 14 modules (similar modules had been merged) were finally screened out by constructing the co-expression matrix (Figure 1D). Moreover, we found that the MEblack module (containing 502 genes) had the highest correlation with the single GSEA score (Cor = 0.61); therefore, it was treated as the key module (Figure 1E).

### 3.2. Functional Pathways Involved in Intersected Genes

Taking the intersection of the DEGs and genes in the key module resulted in 175 intersected genes (Figure 1F). Obviously, intersected genes were involved in GO entries such as ‘mononuclear cell differentiation’, ‘regulation of leukocyte cell–cell adhesion’, ‘lymphocyte differentiation’, ‘regulation of T cell activation’, ‘leukocyte mediated immunity’ (Figure 1G). Meanwhile, they were also associated with ‘osteoclast differentiation-Homo sapiens (human)’, ‘primary immunodeficiency-Homo sapiens (human)’, ‘B cell receptor signaling pathway-Homo sapiens (human)’, ‘chemokine signaling pathway-Homo sapiens (human)’, ‘T cell receptor signaling pathway-Homo sapiens (human)’, and other KEGG pathways (Figure 1H).

### 3.3. Screening of Critical Genes

**Figure 2:**
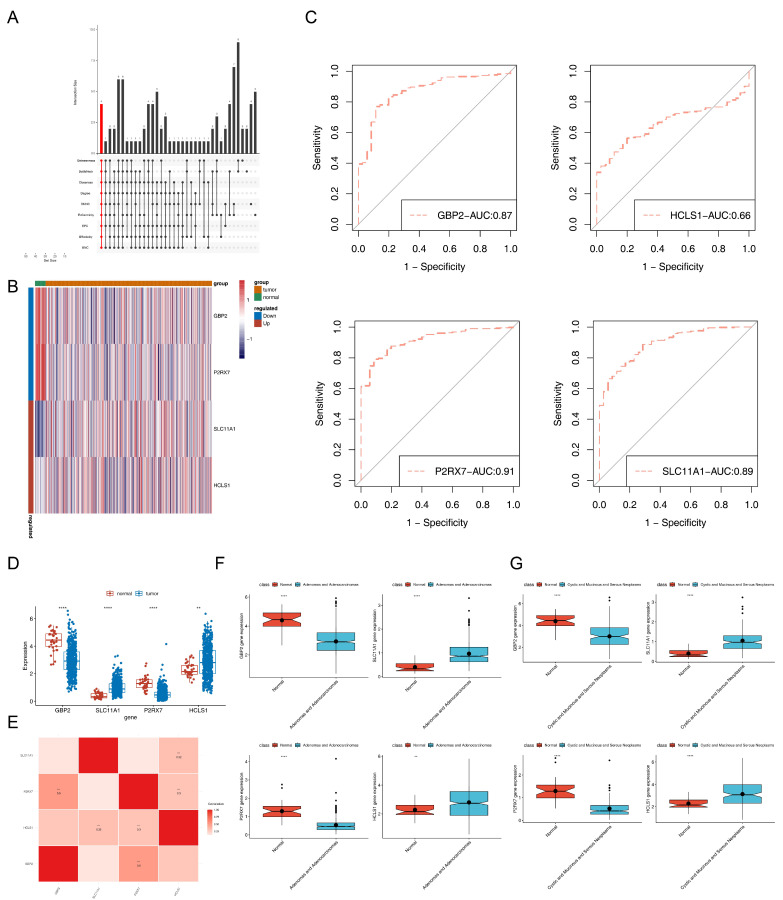
Selection of critical genes and functional annotation analysis of them. (**A**) Selection of critical genes through ten algorithms. The upper bar chart shows the size of the intersection of the top 40 genes ranked by 10 network topology scoring algorithms, with red representing the number of intersection genes among the top 40 genes from all algorithms. The numbers on the bars represent the number of overlapping genes in different sets. (**B**) Heat map of the expression of critical genes in each sample. (**C**) Receiver operating characteristic curves of *P2RX7*, *SLC11A1*, *GBP2*, and *HCLS1* in the Cancer Genome Atlas-EC dataset. The x-axis represents the false positive rate (1-Specificity), and the y-axis represents the true positive rate (Sensitivity). The dashed line denotes the ROC curve corresponding to the *HCLS1* gene, with AUC being the area under the curve. (**D**) Expression of critical genes between tumor and normal samples in the Cancer Genome Atlas-EC dataset (** *p* < 0.01, **** *p* < 0.001). (**E**) Correlation heatmap of the critical genes. The redder the color, the stronger the positive correlation (*** *p* < 0.001). (**F**) Differential expression of the critical genes between the “Adenomas and Adenocarcinomas” subtype group and the normal group (** *p* < 0.01, **** *p* < 0.001). Black circles represent the expression levels of genes in individual samples. (**G**) Differential expression of the critical genes between the “Cystic, Mucinous and Serous Neoplasms” subtype group and the normal group. (**** *p* < 0.001). Black circles represent the expression levels of genes in individual samples.

The 175 intersected genes and 980 reciprocal relationship pairs formed the PPI network, such as FOXP3-MMP9, EMR1-MRC1, PDCD1-CXCL13, and others (Figure S1). Through ten algorithms in the Cytohubba software, we finally identified the four following critical genes: *GBP2*, *SLC11A1*, *P2RX7*, and *HCLS1* (Figure 2A). The heat map demonstrated the expression of the critical genes in each sample (Figure 2B). Consequently, area under the curve (AUC) values of *P2RX7* (AUC = 0.91), *SLC11A1* (AUC = 0.89), *GBP2* (AUC = 0.87), and *HCLS1* (AUC = 0.66) in the TCGA-EC dataset exceeded 0.65, indicating that they had predictive power for EC patients (Figure 2C). Moreover, there were significant differences in the expression of critical genes between EC and normal samples in the TCGA-EC dataset (Figure 2D). The results based on the GSE17025 validation set were shown in Figures S2 and S3. Among the critical genes, the correlation coefficient between *GBP2* and *P2RX7* was 0.5, and that between *HCLS1* and *SLC11A1* was 0.32 (both *p* < 0.001) (Figure 2E). Furthermore, compared with the normal group, the expression trends of key genes in the two subtypes showed significant consistency (*p* < 0.01). Specifically, *GBP2* and *P2RX7* were significantly downregulated in both the “Adenomas and Adenocarcinomas” subtype and the “Cystic, Mucinous and Serous Neoplasms” subtype (*p* < 0.001) (Figure 2F), while *SLC11A1* and *HCLS1* were significantly upregulated in these two subtypes (*p* < 0.01) (Figure 2G).

### 3.4. Functional Annotation Analysis of Critical Genes

**Figure 3:**
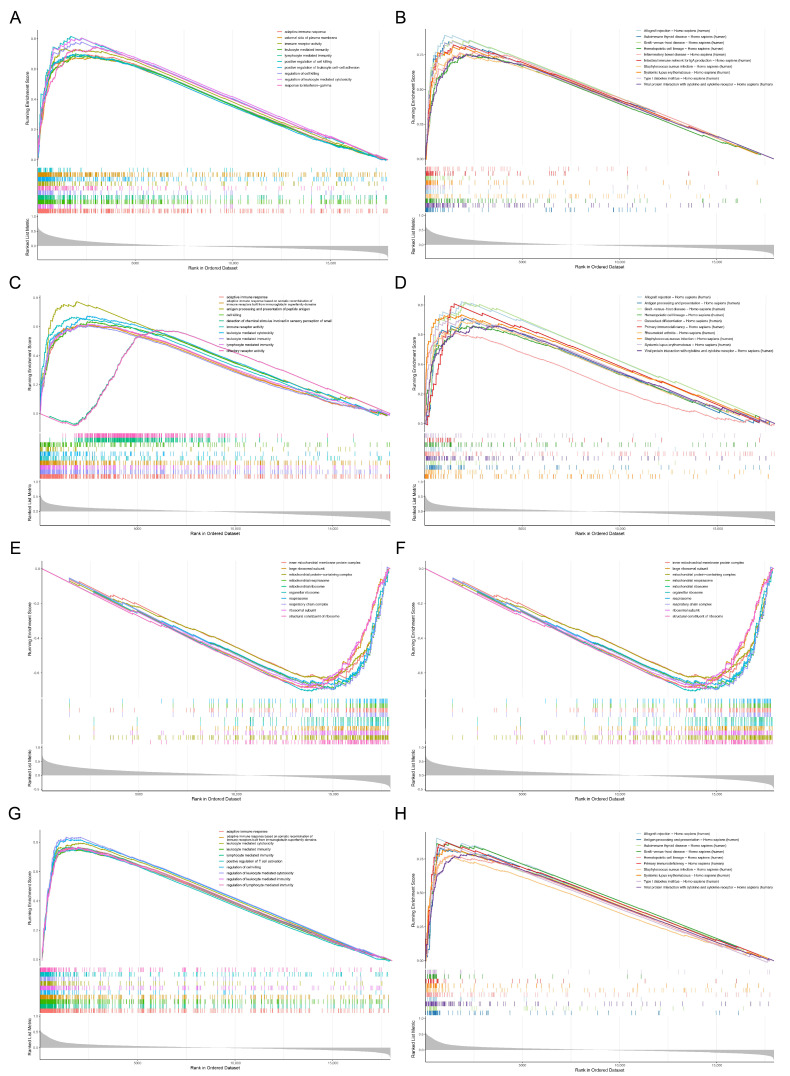
(**A**) GSEA GO results of *GBP2*. The enrichment curves of mitochondria- and ribosome-related gene sets are displayed. The x-axis represents the rank of genes in the ordered dataset (Rank in Ordered Dataset), and the y-axis represents the running enrichment score (Running Enrichment Score). The colored vertical lines in the middle indicate the distribution positions of genes in the gene sets. The gray curve at the bottom shows the background distribution trend of gene set enrichment, which is used to evaluate the significance of gene set enrichment in biological processes. (**B**) GSEA GO results of *HCLS1*. (**C**) GSEA GO results of *P2RX7*. (**D**) GSEA GO results of *SLC11A1*. (**E**) GSEA KEGG results of *GBP2*. (**F**) GSEA KEGG results of *HCLS1*. (**G**) GSEA KEGG results of *P2RX7*. (**H**) GSEA KEGG results of *SLC11A1*.

*GBP2* was enriched to functional pathways, such as ‘regulation of leukocyte mediated cytotoxicity (GO)’, ‘response to interferon-gamma (GO)’, ‘positive regulation of cell killing (GO)’, ‘viral protein interaction with cytokine and cytokine receptor-Homo sapiens (human) (KEGG)’, ‘hematopoietic cell lineage-Homo sapiens (human) (KEGG)’, ‘graft-versus-host disease-Homo sapiens (human) (KEGG)’ (Figure 3A,B). *SLC11A1* was associated with ‘detection of chemical stimulus involved in sensory perception of smell (GO)’, ‘leukocyte mediated cytotoxicity (GO)’, ‘olfactory receptor activity (GO)’, ‘allograft rejection-Homo sapiens (human) (KEGG)’, ‘antigen processing and presentation-Homo sapiens (human) (KEGG)’, ‘rheumatoid arthritis-Homo sapiens (human) (KEGG)’, etc. (Figure 3C,D). The functional pathways in which *P2RX7* was involved contained ‘large ribosomal subunit (GO)’, ‘organellar ribosome (GO)’, ‘mitochondrial ribosome (GO)’, ‘ribosome-Homo sapiens (human) (KEGG)’, ‘oxidative phosphorylation-Homo sapiens (human) (KEGG)’, ‘hematopoietic cell lineage-Homo sapiens (human) (KEGG)’, etc. (Figure 3E,F). *HCLS1* was engaged in functional pathways such as ‘leukocyte mediated cytotoxicity (GO)’, ‘regulation of lymphocyte mediated immunity (GO)’, ‘adaptive immune response (GO)’, ‘primary immunodeficiency-Homo sapiens (human) (KEGG)’, ‘type I diabetes mellitus-Homo sapiens (human) (KEGG)’, ‘systemic lupus erythematosus-Homo sapiens (human) (KEGG)’ (Figure 3G,H).

### 3.5. Cluster Classification of EC Samples

**Figure 4:**
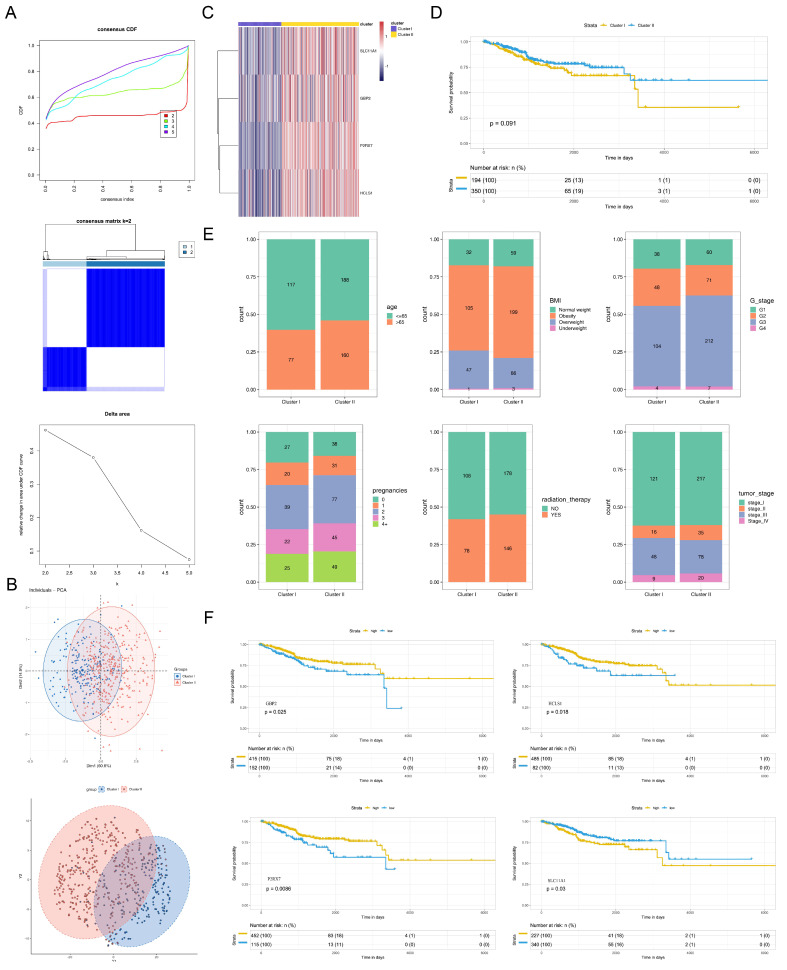
Cluster classification of EC samples and predictive ability of critical genes. (**A**) Consensus clustering analysis. (**Upper**): Comparison plot of cumulative distribution functions (CDF) for continuous value distributions. The x-axis represents continuous values, and the y-axis represents cumulative distribution probabilities (CDF). Curves of different colors correspond to different group sizes. (**Middle**): Visualization of the consensus matrix heatmap, where the color intensity reflects the degree of clustering consistency. (**Lower**): Area under the CDF curve plot for consensus clustering. The x-axis represents k values, and the y-axis represents Delta area index values, which reflect the quantitative results of clustering stability or structural differences with changes in k values and are used to determine the optimal clustering k value. (**B**) Principal component analysis (PCA) and t-distributed stochastic neighbor (t-SNE) embedding analysis. The PCA plot shows the distribution of samples in the two clusters, reflecting the distinguishability and population structure between different clusters. The t-SNE plot displays the distribution of samples in the two clusters, analyzing the tightness within different clusters and the distinguishability between subtypes. (**C**) Heat map of the expression of critical genes in each sample of clusters. (**D**) Kaplan–Meier curves of two clusters. (**E**) Differences in clinical characteristics (stage, tumor stage, age, body mass index, radiation therapy, and pregnancies) between cluster 1 and cluster 2. (**F**) Kaplan–Meier curves of *GBP2*, *SLC11A1*, *P2RX7*, and *HCLS1* between high/low expression groups.

The EC samples were divided into two clusters via consensus clustering analysis (Figure 4A). Afterward, PCA and t-SNE revealed that cluster 1 and cluster 2 were distinguished clearly (Figure 4B). The heat map demonstrated the expression of critical genes in each sample of clusters (Figure 4C). As seen from the Kaplan–Meier (K-M) curve, although there was no significant difference in survival between two clusters (*p* = 0.091), the survival of patients in cluster 2 was slightly better than that of patients in cluster 1 (Figure 4D). Undoubtedly, Figure 4E showed the proportional distribution in different stages of clinicopathological features between cluster 1 and cluster 2.

### 3.6. Predictive Ability of Critical Genes

**Figure 5:**
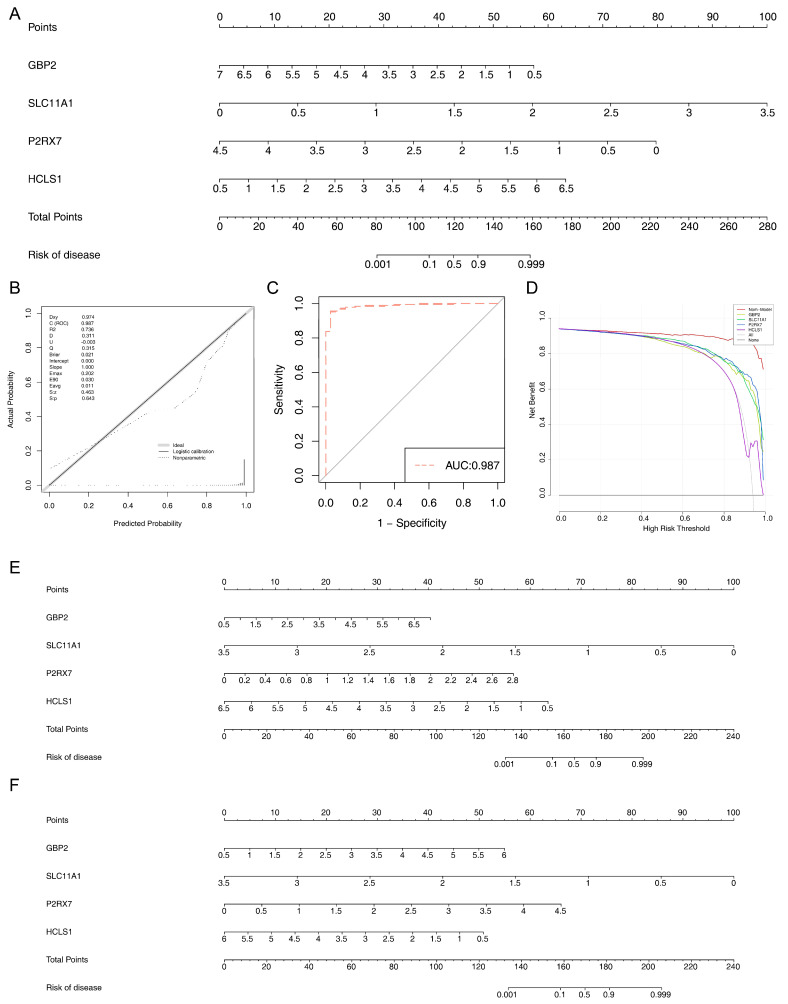
(**A**) EC risk prediction nomogram. It shows the risk score contribution (points dimension) of 4 key genes, namely *GBP2*, *SLC11A1*, *P2RX7*, and *HCLS1*. The “Total Points” below summarizes the total risk score, and the “Risk of disease” at the bottom presents the corresponding distribution of disease risk probability, which is used to quantify the predictive effect of the key gene combination on the risk of endometrial cancer onset. (**B**) Calibration curve. The x-axis represents the predicted probability, and the y-axis represents the actual probability. It shows the fitting of logistic calibration (solid line), non-parametric (dashed line), and ideal calibration (gray). Model evaluation indicators (Dxy, C (ROC), etc.) are listed in the upper right corner, which is used to verify the consistency between the predicted probability of the model and the actual probability of disease occurrence. (**C**) ROC curve of EC risk prediction nomogram. (**D**) Decision curve analysis curves of different models. It shows the changing trends of survival rates with the adjustment of high-risk thresholds when individual key genes and the nomogram model are used as risk indicators for EC. It is used to screen the optimal critical value for risk stratification and assist in disease prognosis evaluation and risk grouping decisions. (**E**) Nomogram for the “Adenomas and Adenocarcinomas” subtype. (**F**) Nomogram for the “Cystic, Mucinous and Serous Neoplasms” subtype.

In the K-M curves, there were significant differences in survival of *GBP2*, *SLC11A1*, *P2RX7*, and *HCLS1* high/low expression groups, and except for *SLC11A1* high/low expression groups, the survival of other critical genes high expression groups outperformed the low expression groups (Figure 4F). The nomogram indicated that the critical genes could effectively predict the prognosis of EC patients (Figure 5A), and the calibration curve further validated the result (Figure 5B). Concurrently, the AUC value of the nomogram was greater than 0.987, indicating that it could predict the prognosis of EC patients extremely well (Figure 5C). The DCA curve illustrated that the benefit rate of the model with critical genes together as the feature was greater than that of the model with individual critical genes as the feature (Figure 5D). In addition, the ROC curve of the model with all the critical genes together was plotted in the validation set with an AUC of 0.920 (Figure S4).

In the nomogram models for the “Adenomas and Adenocarcinomas” and “Cystic, Mucinous and Serous Neoplasms” subtypes, *SLC11A1* exhibited the highest contribution (Figure 5E,F). The calibration curves showed that the slope of the nomogram’s prediction curve was close to 1, indicating that the nomogram had high accuracy in predicting the disease risk of different subtypes (Figure S5A,B). Furthermore, the AUC values of the ROC curves for both nomograms exceeded 0.7, with the AUC value of 0.987 for the “Adenomas and Adenocarcinomas” subtype nomogram (Figure S5C) and 0.991 for the “Cystic, Mucinous and Serous Neoplasms” subtype nomogram (Figure S5D).

### 3.7. Targeted Therapies Against Critical Genes May Have a Considerable Impact on EC

**Figure 6:**
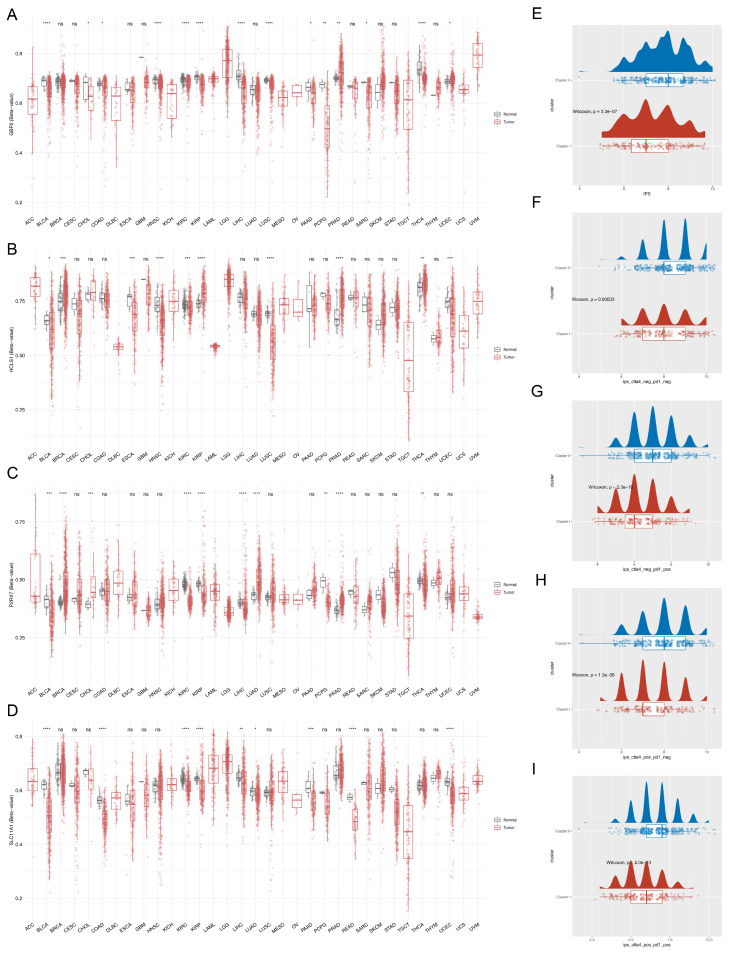
Analysis of CpG methylation and response to immunotherapy. (**A**–**D**) Differences in methylation changes in *GBP2*, *HCLS1*, *P2RX7*, and *SLC11A1* between tumor and normal samples. (**E**–**I**) Differences in immunotherapy scores of PD-1, PD-L1, and CTLA-4 between the two clusters of EC patients (* *p* < 0.05, ** *p* < 0.01, *** *p* < 0.001, **** *p* < 0.001, ns *p* > 0.05).

Following examination of intergroup variations in CpG methylation, it was discovered that a range of malignancies had distinct CpG methylation patterns in critical genes. Notably, there were substantial differences in *GBP2*, *SLC11A1*, and *HCLS1* between EC and normal samples, demonstrating that the control of EC progression is probably influenced by changes in the methylation of critical genes (Figure 6A–D). Meanwhile, to measure the severity of the immune response to EC treatment targeting critical genes, we discovered that all critical genes were positively connected with immunosuppressive factors such as TIGIT, PDCD1, and LAG3, as well as TNF-family stimulatory factors in EC. Our association analysis of critical genes with MHC molecules indicated positive connections with most of the HLA molecules (Figure S6). Finally, TCIA analysis indicated substantial differences in the PD-1, PD-L1, and CTLA-4 immunotherapy ratings between the two clusters (Figure 6E–I). This series of findings suggests that our targeted medicines against critical genes could have a significant impact on EC.

### 3.8. Immunity Microenvironment Analysis

**Figure 7:**
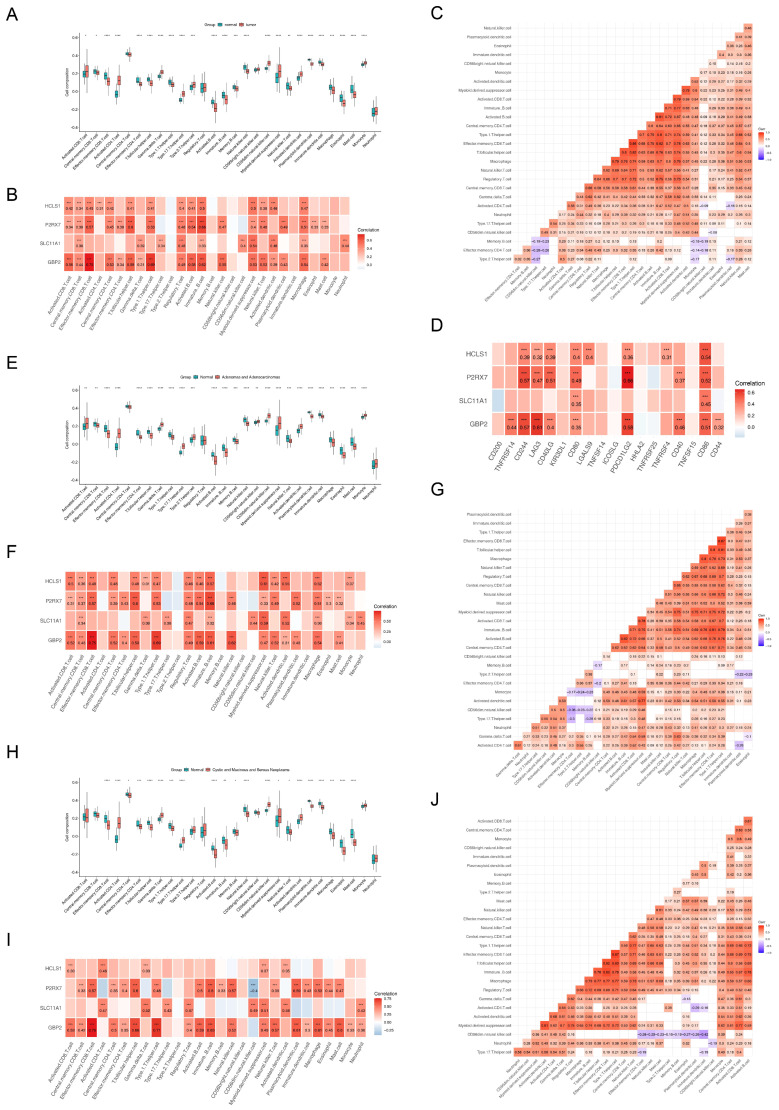
Immunity microenvironment analysis (* *p* < 0.05, ** *p* < 0.01, *** *p* < 0.001, **** *p* < 0.001). (**A**) Differences in immune cell infiltration abundance between tumor and normal samples (**B**) Correlations between critical genes and immune cells. (**C**) Correlations among immune cells. (**D**) Correlations between critical genes and immune checkpoints. (**E**) Differences in immune cell composition between the “Adenomas and Adenocarcinomas” subtype and the normal group. (**F**) Correlation heatmap between immune cells and critical genes: the redder the color, the stronger the positive correlation; the bluer the color, the stronger the negative correlation. (**G**) Correlations among immune cells. (**H**) Differences in immune cell composition between the “Cystic, Mucinous and Serous Neoplasms” subtype and the normal group. (**I**) Correlation heatmap between immune cells and critical genes: the redder the color, the stronger the positive correlation; the bluer the color, the stronger the negative correlation. (**J**) Correlations among immune cells.

A total of 24 kinds of immune cells were significantly different in infiltration level between tumor and normal samples, except for central memory CD4 T cells, regulatory T cells, CD56 bright natural killer cells, and neutrophils (Figure 7A). After that, we found that there were positive correlations between all critical genes and most immune cells, of which *GBP2* had the highest correlation with effector memory CD8 T cells (Cor = 0.75, *p* < 0.001) (Figure 7B). Ultimately, we also found that effector memory CD8 T cells had the strongest positive correlation with type 1 helper T cells (Cor = 0.86) (Figure 7C). Moreover, *P2RX7* had the highest correlation with the PDCD1LG2 immune checkpoint (Cor = 0.66, *p* < 0.001) (Figure 7D).

In the “Adenomas and Adenocarcinomas” subtype and the normal group, there were extensive and significant differences in the composition of 25 types of immune cells (*p* < 0.01) (Figure 7E). Taking CD8^+^ T cell subsets as an example, compared with the normal group, the proportion of effector memory CD8^+^ T cells in the “Adenomas and Adenocarcinomas” subtype was significantly increased, while the proportions of central memory CD8^+^ T cells and activated CD8^+^ T cells were significantly decreased. This suggested that the subtype might have an immune characteristic of “effector memory differentiation” of CD8^+^ T cells, which might be associated with long-term tumor immune surveillance. Meanwhile, effector memory CD8^+^ T cells also showed the strongest positive correlation with *GBP2* (cor = 0.75, *p* < 0.05) (Figure 7F) and the highest positive correlation with type 1 T helper cells (cor = 0.87) (Figure 7G).

In the “Cystic, Mucinous and Serous Neoplasms” subtype and the normal group, there were differential infiltrations of 21 types of immune cells (*p* < 0.05). Among these immune cells, compared with the normal group, the proportions of effector memory CD8^+^ T cells, type 1 T helper cells, and other immune cells in the subtype were significantly decreased, while the proportion of activated CD4^+^ T cells and other immune cells was significantly increased (*p* < 0.05) (Figure 7H). The correlation analysis showed consistent results with those of the “Adenomas and Adenocarcinomas” subtype: effector memory CD8^+^ T cells had the strongest correlation with *GBP2* (cor = 0.76, *p* < 0.001) (Figure 7I), and the strongest correlation with type 1 T helper cells (cor = 0.87) (Figure 7J).

### 3.9. Sensitivity Analysis of Chemotherapeutic Agents

**Figure 8:**
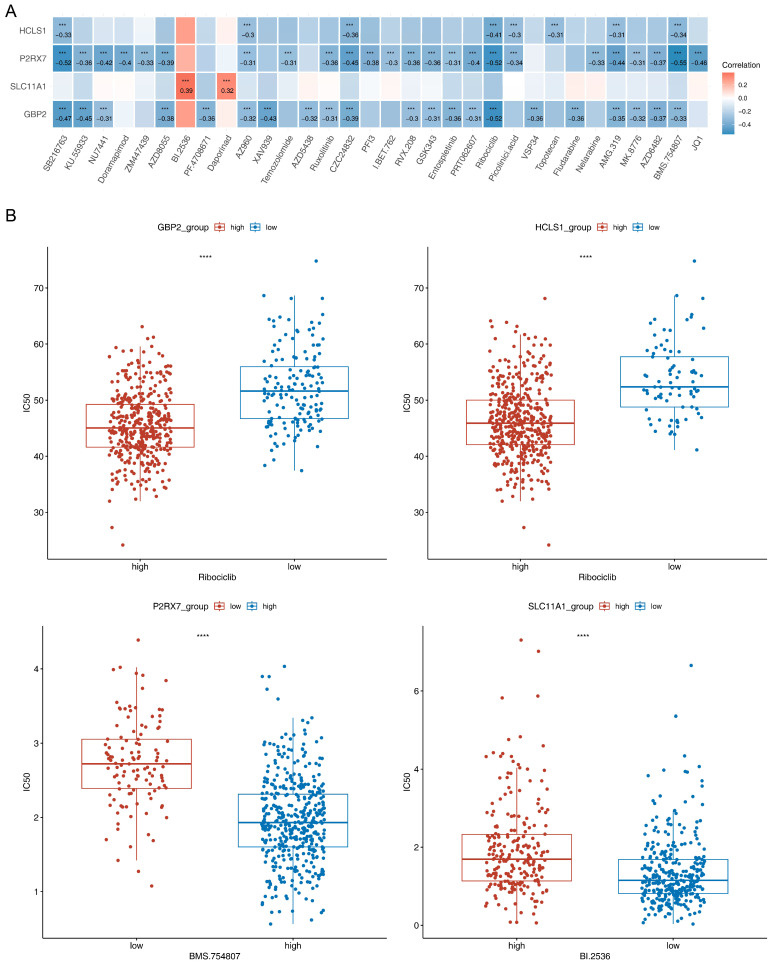
The sensitivity analysis of chemotherapeutic agents. (**A**) Heat map of correlations between critical genes and chemotherapeutic agents. (**B**) Differences in the IC50 of Ribociclib, BI.2536, and BMS.754807 between critical genes high/low expression groups (*** *p* < 0.001, **** *p* < 0.001).

Through Spearman’s correlation analysis, we found the highest passive correlation of *HCLS1* (Cor = −0.41, *p* < 0.001) and *GBP2* (Cor = −0.52, *p* < 0.001) with “Ribociclib”, as well as *P2RX7* with “BMS.754807” (Cor = −0.55, *p* < 0.001). Whereas *SLC11A1* was the most strongly positively connected with “BI.2536” (Cor = 0.39, *p* < 0.001) (Figure 8A). In addition, the IC_50_ of these drugs were all notably different between related critical genes high/low expression groups (*p* < 0.0001) (Figure 8B). The patients with high expression of *HCLS1*, *GBP2*, and *P2RX7* were more susceptible to “Ribociclib” and “BMS.754807”, while patients with high expression of *SLC11A1* were more tolerant of “BI.2536”.

### 3.10. Validation of Critical Genes Expression by Immunochemistry and qRT-PCR

**Figure 9:**
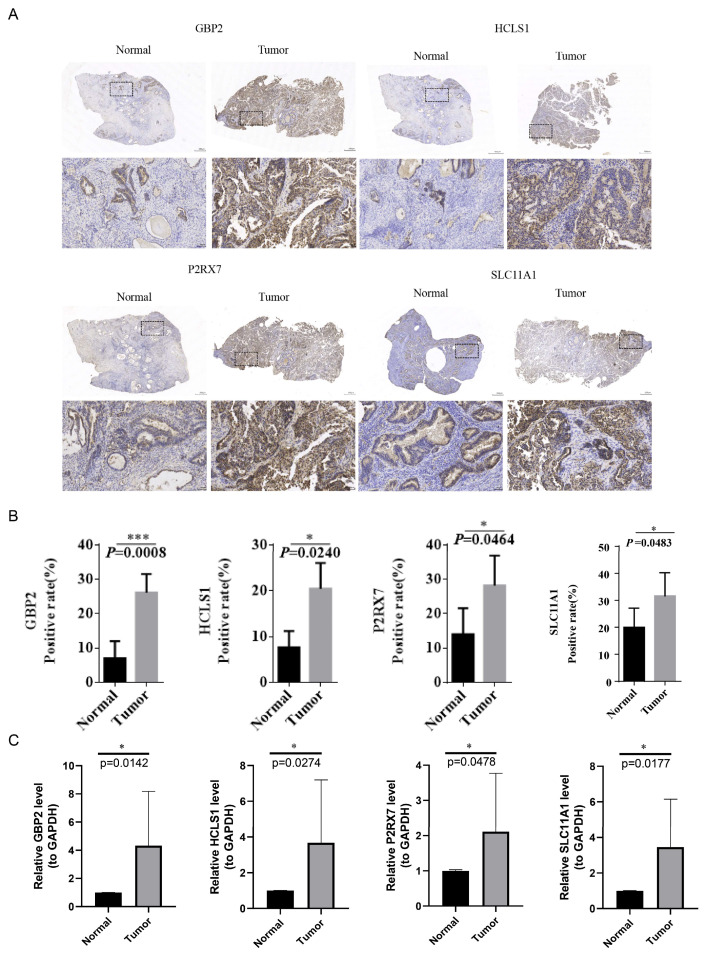
Validation of critical genes by immunochemistry and quantitative real-time polymerase chain reaction. (**A**) Representative immunochemistry staining of *GBP2*, *HCLS1*, *P2RX7*, and *SLC11A1* among 10 tumor and normal samples. The magnification is full field of view, 200×, and the scale is 50 μm. (**B**) The immunochemistry staining positive rate (%) of *GBP2*, *HCLS1, P2RX7*, and *SLC11A1* between normal and tumor groups. (**C**) Relative mRNA expression level (to GAPDH) of *P2RX7*, *SLC11A1*, *GBP2*, and *HCLS1* between normal and tumor groups (* *p* < 0.05, *** *p* < 0.001).

The results of immunochemistry showed that *GBP2*, *HCLS1*, *P2RX7*, and *SLC11A1* were more highly expressed in the tumor group than in the normal group (Figure 9A,B). The results of qRT-PCR showed that these four critical genes were significantly up-regulated in the tumor group compared to the normal group (Figure 9C). Generally, the expression trends of these genes were consistent with the result of bioinformatic analysis.

## 4. Discussion

As the fourth most common cancer in women, EC patients are primarily treated with surgery [1]. The prognoses of EC are related to tumor stage, pathological type, tumor differentiation, and so on. The advanced and recurrent EC patients have poorer outcomes [1], and subsequent treatment options may be limited and ineffective for some of the patients. Consequently, there is an urgent need to find new biomarkers to evaluate the prognosis of EC earlier and more effectively, as well as to discover new treatments for EC patients. Accumulating evidence has shown that RCD is highly correlated with tumorigenesis. Different pathways in RCD will affect the development of cancer and the response towards treatment [18]. Through regulating multiple RCD signal pathways by a drug or gene, the drug resistance of cancer cells to a specific type of RCD can be avoided [18]. Analyzing the roles of RCD in the human body, especially in tumor cells, enables us to better understand intracellular signal molecules, maintenance of homeostasis, and mechanisms of drug resistance. Therefore, exploring different RCD pathways may be a new direction of cancer studies in the future. However, there is still no specific conclusion on the role of RCD in EC. This study identified four critical RCD-RGs, namely *GBP2*, *SLC11A1*, *P2RX7*, and *HCLS1*, via bioinformatics analyses, and a prediction model was constructed. The relationships between key genes and various immune features were also deeply explored, and targeted drugs were identified. These conclusions may serve as new directions for future research in EC.

It is worth noting that the key genes we identified in this study (*GBP2*, *SLC11A1*, *P2RX7*, and *HCLS1*) are different from those previously reported to be associated with RCD in EC. Most of the previous studies focused on single RCD type genes, for example, ferroptosis-related genes (such as *ATF4*)- which mainly regulate lipid peroxidation [19] -or apoptosis-related genes (such as *TERT*)- whose dysregulation is associated with cisplatin resistance [20]. In contrast, the selected key genes in this study uniquely participate in immune regulation and the cross-talk between RCD and the immune microenvironment. Moreover, previous studies have mostly constructed prognostic models based on a single RCD type (such as ferroptosis) [21], while the model in our study integrated multiple RCD-related genes and demonstrated superior predictive performance (AUC = 0.987 in the TCGA-EC dataset). Additionally, each gene can also effectively predict the survival of EC patients, suggesting that it can reflect the progression of EC more comprehensively. Last but not least, *SLC11A1* and *HCLS1* have rarely been reported in studies in EC. This study found that the above two genes were related to the sensitivity to chemotherapy drugs (such as *HCLS1* and Ribociclib) and immune checkpoint molecules, providing a new direction for traditional RCD pathway studies in EC.

*GBP2* belongs to the dynamin superfamily of large GTPases, famous for its cell-autonomous immunity against microbial pathogens, inflammation, and cancer development [22,23]. In this study, we firstly found that *GBP2* had the highest correlation with effector memory CD8 T cells (Cor = 0.75, *p* < 0.001). This suggests that *GBP2* may regulate the progression of EC by enhancing the retention of antigen-specific T cells, which is completely different from its antibacterial effect. Meanwhile, CD8^+^ T cells can induce tumor cell apoptosis by releasing granzyme B [24]. This correlation indicates that *GBP2* may directly promote the apoptosis and clearance of EC cells by enhancing the apoptotic signal mediated by T cells [24]. Moreover, Godoy et al. [25] found that when *GBP2* was overexpressed in breast cancer, the prognosis was significantly better, and the upregulation of *GBP2* also indicated efficient T cell response. This conclusion was consistent with our finding. *GBP2* enhances paclitaxel sensitivity in triple-negative breast cancer by promoting autophagy in combination with ATG2 and inhibiting the PI3K/AKT/mTOR pathway [26]. Wang et al. [22] confirmed that *GBP2* could regulate PD-L1 expression via STAT1 signaling in colorectal cancer, and patients with high *GBP2* expression can have a better response to anti-PD-1 therapy [22]. Studies have shown that the infection of microorganisms may lead to chronic inflammation, such as endometritis and pelvic inflammatory disease [27]. This greatly increases the risk of EC [27]. A possible mechanism is that bacterial toxins activate the production of tumor-promoting metabolites, which then lead to dramatic change in the activity of immune cells and the expression of inflammatory genes. This shows that the overexpression of *GBP2* may induce RCD in EC by affecting microorganisms and immunoactive cells (for example, by increasing T cell response and migration or by activating inflammation in the process).

*SLC11A1*, known as natural resistance-associated macrophage protein-1, is a member of the solute-carrier family [28]. It can be recruited to the phagosomal membrane after phagocytosis and regulates the host resistance and susceptibility to a range of pathogens [28]. In immune cells, *SLC11A1* influences the major histocompatibility complex class II expression and antigen-presenting cell function [28] and plays a role in innate immunity, autoimmune diseases, and infection [29,30,31]. In patients with glioma, *SLC11A1* was identified as a stratification indicator for immunotherapy or chemotherapy [32]. *SLC11A1* was also associated with immune reduction in metastatic melanoma patients treated with targeted therapy [33]. Moreover, *SLC11A1* has been suggested to be included in a prognosis signature and used for potential therapeutic drug prediction for renal clear cell carcinoma [34,35]. Finally, *SLC11A1* has been linked to the efficacy of immunotherapy in colorectal cancer [28]. However, its role in EC exhibits unique characteristics. Although *SLC11A1* shows excellent immunological prognostic value in other cancers, our K-M curves show better survival for high-expression groups of *GBP2*, *P2RX7*, and *HCLS1* but not *SLC11A1* in EC. This result indicates that the immunoregulatory function of *SLC11A1* in EC may be weakened, which might be related to the unique hormonal microenvironment of EC. It is known that estrogen is a key driver of EC, and it can cause the polarization of intratumoral macrophages towards a tumor-promoting phenotype [36]. This might weaken the role of *SLC11A1* in enhancing innate immunity. We have also found that *SLC11A1* was associated with chemotherapeutic agent sensitivity in EC. The gene was strongly positively connected with the drug “BI.2536”. BI.2536, a polo-like kinase 1 inhibitor, was proved to trigger mitotic catastrophe (a type of RCD) by inducing mitotic arrest [37]. This suggests that in EC, *SLC11A1* may affect the mitotic catastrophe process in RCD by regulating cell cycle checkpoints. This is quite different from the mechanism by which *SLC11A1* indirectly affects RCD through immunoregulation in other cancers. This finding may support the functional positioning of *SLC11A1* as an RCD-related gene in EC. According to the evidence displayed above, the role of *SLC11A1* in EC is not a simple extension of its function in other cancers but has adapted to the unique pathological environment (hormonal microenvironment, unique treatment vulnerability) of EC. Further research is needed to clarify how estrogen signaling or mitotic regulatory pathways interact with *SLC11A1* to affect the progression of EC.

The *P2RX7* receptor is an ATP-gated ion channel, mainly responsible for forming ion channels and water pores [38]. In the microenvironment of tumor growth, extracellular high levels of ATP activate *P2RX7*. Then *P2RX7* participates in cell proliferation and tumor metastasis by inducing cells to secrete cell cytokines such as interleukin-6 [39]. In breast cancer cell lines of MCF-7 and MDA-MB-231, increasing *P2RX7’s* expression in tumor cells maintains tumor cell survival and invasion ability by activating ERK1/2 and Akt pathways [40]. In gastric cancer cells, *P2RX7* enhanced the proliferation, migration, and invasion of cancer cells via modulating ERK1/2 and Akt pathways and epithelial–mesenchymal transition [39]. However, in some other tumors or cell lines, *P2RX7* worked as a tumor suppressor. In human colon tumor HCT-8 and Caco-2 cell lines or in glioma cell line GL261, the activation of *P2RX7* could inhibit tumor cell proliferation [41,42]. This may be because the mechanism of the role of *P2RX7* in oncogenesis is more complicated than we expected. A study [43] showed that *P2RX7* expression is lesser in EC cells compared to normal cells. Decreased *P2RX7* expression and the abrogated *P2RX7*-mediated apoptosis predispose proliferative endometrial cells to the effects of carcinogens and may lead to the development of EC [43]. This conclusion is consistent with our finding that the prognoses of EC patients are better in the *P2RX7* high expression group than those in the low expression group. Based on the results mentioned above, we reasonably hypothesize that dysregulation of *P2RX7* may mediate inflammasome activation cytokine and chemokine release, resulting in RCD in EC.

*HCLS1*, a substrate of antigen receptor-coupled tyrosine kinase, is pivotal in lymphoid cell antigen receptor signaling, influencing clonal expansion and deletion [44]. *HCLS1* controls the process of cellular viability, migration, and cancer progression [44]. Yuan et al. [45] found that the overexpression of *HCLS1* can reduce the ability of proliferation, migration, and invasion in cancer cells and significantly suppress tumor growth in vivo. *HCLS1* was also upregulated in high-grade primary tumors with high plasma cell content, which were associated with increased predicted response towards immunotherapy [45]. In addition, when subjected to oxidative stress, *HCLS1*-overexpressing cells exhibit an enhanced autophagy induction. The *HCLS1*-related protein HAX1 can regulate the process of autophagy through specific domains [46], and autophagy, one of the RCD types, is then associated with the survival and proliferation of EC cells [47]. This suggests that *HCLS1* may influence EC progression by affecting tumor cell proliferation and metastasis.

Targeted therapies or immunotherapies against these four critical genes may have a considerable impact on EC [48,49]. Compared with the normal group, we found the expression trends of key genes in the two subtypes showed significant consistency. *GBP2* and *P2RX7* were significantly downregulated in both subtypes, while *SLC11A1* and *HCLS1* were significantly upregulated in these two subtypes. This consistency indicates that these four genes target the core malignant phenotypes of EC (such as RCD deficiency and immune escape) rather than subtype-specific characteristics. The stability of the subtype-specific nomogram further confirms this. On the other hand, the silencing of tumor suppressor genes by promoter CpG island methylation is an important cause of oncogenesis. For example, two famous oncogenic events, silencing of *MLH1* and *BRCA1*, result from promoter CpG methylation [50]. In our study, we also discovered distinct CpG methylation patterns in critical genes between EC and normal tissues. This raises the perspective that aberrant methylation could be causative of genomic instability and malignant transformation in EC. As alterations in aberrant methylation are relatively stable and may be reversible therapeutically [51], we plan to further validate these methylation markers using methylation-specific PCR technology and can exploit it to define more precise and effective molecular targets in gene-based therapies for EC in the future. As for immunity, all critical genes were positively correlated with various immunosuppressive factors as well as TNF-family stimulatory factors in EC. Patients with high expression of critical genes showed better immune response towards PD-1, PD-L1, and CTLA-4 immunotherapy. Additionally, 24 immune cell species, including central memory CD4 T cells, regulatory T cells, and so on, were found to play an essential role in the EC microenvironment, most of which were correlated positively with critical genes. Detecting potential immune checkpoints, according to our findings, can also offer novel direction of immunotherapies for EC. *GBP2* had the highest correlation with effector memory CD8 T cells, and *P2RX7* had the highest correlation with the PDCD1LG2 immune checkpoint. Effector memory CD8 T cells play important roles in tumor immunity and protection based on their ability to clonally expand and exert cytotoxic function [52]. The accumulation and retention of CD8 T cells are likely a key component of a beneficial immune response and are associated with a prolonged survival in EC [53]. Immune checkpoint PDCD1LG2 inhibits the activation of T cells by binding to PD-1 receptors, thus protecting tumors from the attack of the immune system [54]. High expression of PDCD1LG2 in EC is associated with low overall survival rate and poor prognosis [55]. To conclude, *GBP2* and *P2RX7* may affect the development of EC through regulating the activity of effector memory CD8 T cells and the expression of PDCD1LG2, respectively.

The patients with high expression of *HCLS1*, *GBP2*, and *P2RX7* were more susceptible to “Ribociclib” and “BMS.754807”, while patients with high expression of *SLC11A1* were more tolerant of “BI.2536”. Ribociclib is a cyclin-dependent kinase (CDK) 4/6 inhibitor [51]. This drug interrupts the growth of malignant cells by inhibiting progression through the cell cycle via the CDK-RB1-E2F pathway in many cancers [51,56,57]. It has been proved to have positive survival effects in relapsed estrogen receptor-positive ovarian cancer and EC, and positive survival effects were observed in patient-derived xenograft models [58]. Therefore, Ribociclib may have promising clinical activity in EC, and further clinical research is still ongoing. BMS.754807, an inhibitor targeting the insulin-like growth factor 1 receptor and insulin receptor family kinases, has been in phase I trials for the treatment of human cancers, including breast, lung, pancreatic, colon, and gastric tumor cell lines [59,60]. Zhang et al. [60] demonstrated that BMS.754807 inhibited lung cancer cell growth in vitro, while it induced autophagy as well as cell cycle arrest at the G1 phase. This drug could suppress the expression of cell cycle marker proteins and the PI3K/Akt/mTOR signaling pathway. Hassan et al. [61] confirmed that nab-paclitaxel, when combined with BMS.754807, enhanced the inhibition of cell proliferation and increment in cell apoptosis in esophageal adenocarcinoma in vitro and in vivo. The drug is very promising for the clinical treatment of tumors in the future. BI.2536, a polo-like kinase 1 inhibitor, was suggested to induce mitotic arrest and a subsequent surge in apoptosis [62,63]. BI.2536 could induce GSDME-dependent pyroptosis concurrent with caspase-3-mediated apoptosis in ovarian cancer [64]. Its safety and effectiveness were also tested in vivo at the same time [64]. BI.2536-induced pyroptosis might have great potential for improving the outcomes of immunotherapy and traditional chemotherapy. BI.2536 could also enhance the transition of EC cells through G2 and abrogate the G2/M checkpoint [65]. The loss of the G2/M checkpoint moves EC cells that are genomically unstable prematurely into M phase, leading to widespread cell death due to mitotic catastrophe [65]. Generally, the above three drugs can inhibit tumor development, which suggests that they can probably be used as potential targeted drugs in the treatment of EC. However, further clinical trials of these drugs in EC are needed.

Furthermore, the K-M curves showed significant differences in survival of *GBP2*, *SLC11A1*, *P2RX7*, and *HCLS1* in high/low expression groups, which indicated that the biomarkers can be used to predict the prognosis of EC. *GBP2* and *SLC11A1* are associated with immunotherapy response and may affect the therapeutic effect by jointly regulating T cell activity. *HCLS1* may have a similar function to *P2RX7* by affecting tumor cell proliferation and metastasis. These genes may have potential synergistic effects with each other. No single marker has been proved to be a panacea. Compared to single prognostic variables, multivariate prediction models can provide more useful complementary information and help improve prognosis accuracy and treatment selection. Multigene prognostic models have already been encouraged to be applied in many cancers, including breast cancer [66], laryngeal cancer [67], and so on. Therefore, we developed the first prognosis prediction nomogram based on RCD-RGs together, and the model showed a satisfactory AUC value. The nomogram has been widely used as a predictive method in oncology in recent years, which can visualize and individualize prediction under different situations [68,69]. Developing a clinically useful nomogram of EC is instrumental in helping clinicians screen out high-risk EC groups better. With earlier diagnosis and targeted personalized treatment, these patients’ prognoses are likely to be improved.

In conclusion, new biomarkers based on RCD were identified in this study via the TCGA database, and the corresponding prediction model was constructed. This provides an important reference for the diagnosis of EC and improvement of patients’ prognoses. As the tumor samples are much easier to acquire, the problem of unbalanced sample distribution is inevitable, which is also very common in many similar studies [7,70]. However, the small sample size may lead to reduced statistical power. This imbalance may cause the model to predict more accurately for tumor samples and less accurately for normal samples, thus affecting the generality and reliability of the results. To minimize the influence of sample size imbalance on our results, this study applied WGCNA, differential expression analysis, and consensus clustering analysis when selecting new biomarkers. Moreover, the TCGA-EC dataset has been widely used in the studies of EC, so the reliability of the data is unquestioned.

This research also carried out qRT-PCR and IHC to validate the expression trends of these biomarkers. Although the sample size was small, this validation was designed to preliminarily confirm the expression trends of the key genes in the bioinformatics analysis. The results showed that the mRNA expression changes in the four key genes were basically consistent with those in the TCGA and GEO databases, providing directional support for the subsequent large-sample validation. In addition, the IHC experiments in this study focused on the validation of the expression differences in key proteins and did not conduct protein-level correlation analysis. In future studies, we will observe the co-localization patterns of *GBP2*/*P2RX7* and *HCLS1*/*SLC11A1* through double-label immunofluorescence and verify the protein interactions through co-immunoprecipitation to clarify the translation consistency of the gene-level synergy at the protein level. We must admit that there are potential problems in the initial sample classification. We can only ensure that the tissues included in the normal group have no potential pathological lesions, but we cannot completely rule out the influence of non-pathological changes. This may lead to the final expression trend not fully reflecting the differences between completely normal tissues and tumor tissues. In our future studies, we will increase the number of normal samples by expanding sample sources or using multicenter datasets to mitigate the impact of sample imbalance on model performance. We will also adopt a prospective sample collection strategy and conduct real-time sample quality control in collaboration with pathologists. In this study, we focused more on genes with high expression levels when selecting key genes, which might result in insufficient coverage of genes with low expression but having important functions in EC. Future research can consider verifying the biological significance of such genes by integrating single-cell RNA sequencing or functional screening to complement the shortcomings of this study. Meanwhile, the AUC value of our nomogram model is 0.987, which may indicate overfitting. Additionally, whether these new biomarkers improve prognostication over other biomarkers or the exact therapeutic effects of the potential drugs in EC remains unclear, as our research lacks the data from clinical institutions. And at present, functional experiments are lacking to confirm mechanistic roles of these critical genes in EC. We will keep up with the research progress of the role of RCD in EC and further carry out functional experiments. By constructing EC cell models with the knockdown/overexpression of critical genes, we will measure the changes in cell apoptosis rate, pyroptosis-related indicators, and ferroptosis markers and clarify different genes’ regulatory effects on specific RCD subtypes. Meanwhile, we will apply RNA-seq to screen differentially expressed downstream target genes and carry out dual-luciferase reporter gene experiments to verify their interaction with key molecules of the RCD pathway, revealing the specific regulatory network. For potential therapeutic drugs, we will select drugs with high sensitivity, such as Ribociclib and BMS.754807. In EC cell lines with high or low expression of key genes, we will detect IC_50_ values and changes in cell viability after drug treatment and apply Western blot to analyze the effects of the drugs on cell cycle and apoptosis-related proteins (such as Cyclin D1 cleaved-caspase 3), preliminarily clarifying the association mechanism between drug sensitivity and gene expression. Subsequently, we will verify the drug’s anti-tumor effect in vivo through nude mouse xenograft models, providing experimental evidence for clinical use. In terms of clinical translation, we plan to collaborate with multiple centers to collect more clinical pathological data and follow-up data of EC patients (encompassing EC patients of different stages and degrees of differentiation). Through K-M analysis and Cox regression models, we will compare the independent value of the key genes identified in this study with existing clinical indicators (such as FIGO staging) in prognosis prediction. We will also use standardized techniques such as qRT-PCR and IHC to verify the diagnostic efficacy of key genes and integrate clinical routine indicators (such as age and BMI) to construct a joint nomogram model to evaluate its feasibility as a clinical decision-making tool.

## 5. Conclusions

RCD-RGs (namely *GBP2*, *SLC11A1*, *P2RX7*, and *HCLS1*) are associated with immune infiltration and multiple functional pathways in EC; they may play a role in EC development. These findings can provide a new perspective for the clinical treatment and prognosis prediction of EC, which has potential clinical value, and can also provide evidence for developing targeted and personalized treatment plans.

## Data Availability

The datasets generated during and/or analyzed during the current study are available from the corresponding author on reasonable request.

## Supplementary Materials

The following supporting information can be downloaded at: https://www.mdpi.com/article/10.3390/biomedicines13092289/s1, Table S1: The clinical characteristics of the samples; Table S2: Eighteen immune checkpoint molecules selected from the literature; Table S3: Primer sequences in quantitative real-time polymerase chain reaction; Table S4: DEGs; Figure S1: The protein–protein interaction network. Nodes represent genes, and edges represent interactions between them; Figure S2: Receiver operating characteristic curves of *P2RX7*, *SLC11A1*, *GBP2* and *HCLS1* based on the GSE17025 validation set; Figure S3: Expression of critical genes between tumor and normal samples based on the GSE17025 validation set; Figure S4: Receiver operating characteristic curve of the nomogram plotted in the GSE17025 validation set; Figure S5: (**A**) Calibration curve of the nomogram for the “Adenomas and Adenocarcinomas” subtype. (**B**) Calibration curve of the nomogram for the “Cystic, Mucinous and Serous Neoplasms” subtype. (**C**) ROC curve of the “Adenomas and Adenocarcinomas” subtype. (**D**) ROC curve of the “Cystic, Mucinous and Serous Neoplasms” subtype; Figure S6: From left to right and top to bottom, they are the Spearman correlation analysis of *GBP2*, *HCLS1*, *P2RX7*, and *SLC11A1* with immunosuppressants, the Spearman correlation analysis of the four genes with immune activators, and the Spearman correlation analysis of these four genes with the major histocompatibility complex, respectively.

## Abbreviations

The following abbreviations are used in this manuscript:

AUC	Area under the curve
CDF	Cumulative distribution function
DCA	Decision curve analysis
DEGs	Differentially expressed genes
EC	Endometrial cancer
GAPDH	Glyceraldehyde-3-Phosphate Dehydrogenase
GEO	Gene Expression Omnibus
GDSC	Genomics of Drug Sensitivity in Cancer
GSEA	Gene set enrichment analysis
GSVA	Gene set variation analysis
GO	Gene ontology
IC_50_	The 50% inhibitory concentration
IPS	Immunophenotypic scores
KEGG	Kyoto encyclopedia of genes and genomes
K-M	Kaplan–Meier
MHC	Major histocompatibility complex
NOS	Not Otherwise Specified
PCA	Principal component analysis
PD-1/PD-L1	Programmed death protein-1/programmed cell death ligand-1
PPI	Protein–protein–network
qRT-PCR	Quantitative real-time polymerase chain reaction
RCD	Regulated cell death
RCD-RGs	Regulated cell death-related genes
ROC	Receiver operator characteristic
SMART	The Shiny Methylation Analysis Resource Tool
ssGSEA	Single-sample gene set enrichment analysis
TCGA	Cancer Genome Atlas
TCIA	Cancer Immunome Atlas
t-SNE	t-distributed stochastic neighbor embedding
WGCNA	Weighted gene co-expression network analysis
