# Supplementary material for: Exploratory Analysis of Regulated Cell Death-Related Genes as Potential Prognostic Biomarkers in Endometrial Carcinoma
Source: Biomedicines. 2025 Sep 17;13(9):2289. doi: 10.3390/biomedicines13092289 (PMC12467941; doi:10.3390/biomedicines13092289)
Supplement: Supplementary file 1 [file biomedicines-13-02289-s001.zip › Table S1.pdf]

Table S1. The clinical characteristics of samples.

Type	Sample count (n)
Neoplasm histologic grade	
G1	98
G2	119
G3	316
G4	11
Tumor stage	
Stage I	338
Stage I	51
Stage I	126
Stage IV	29
Radiation therapy	
Yes	224
No	286
Body mass index	
Underweight	4
Normal weight	91
Overweight	113
Obesity	304
Pregnancies	
0	65
1	51
2	116
3	67
4（+）	74
