# Supplementary figures and images for: Exploratory Analysis of Regulated Cell Death-Related Genes as Potential Prognostic Biomarkers in Endometrial Carcinoma
Source: Biomedicines. 2025 Sep 17;13(9):2289. doi: 10.3390/biomedicines13092289 (PMC12467941; doi:10.3390/biomedicines13092289)

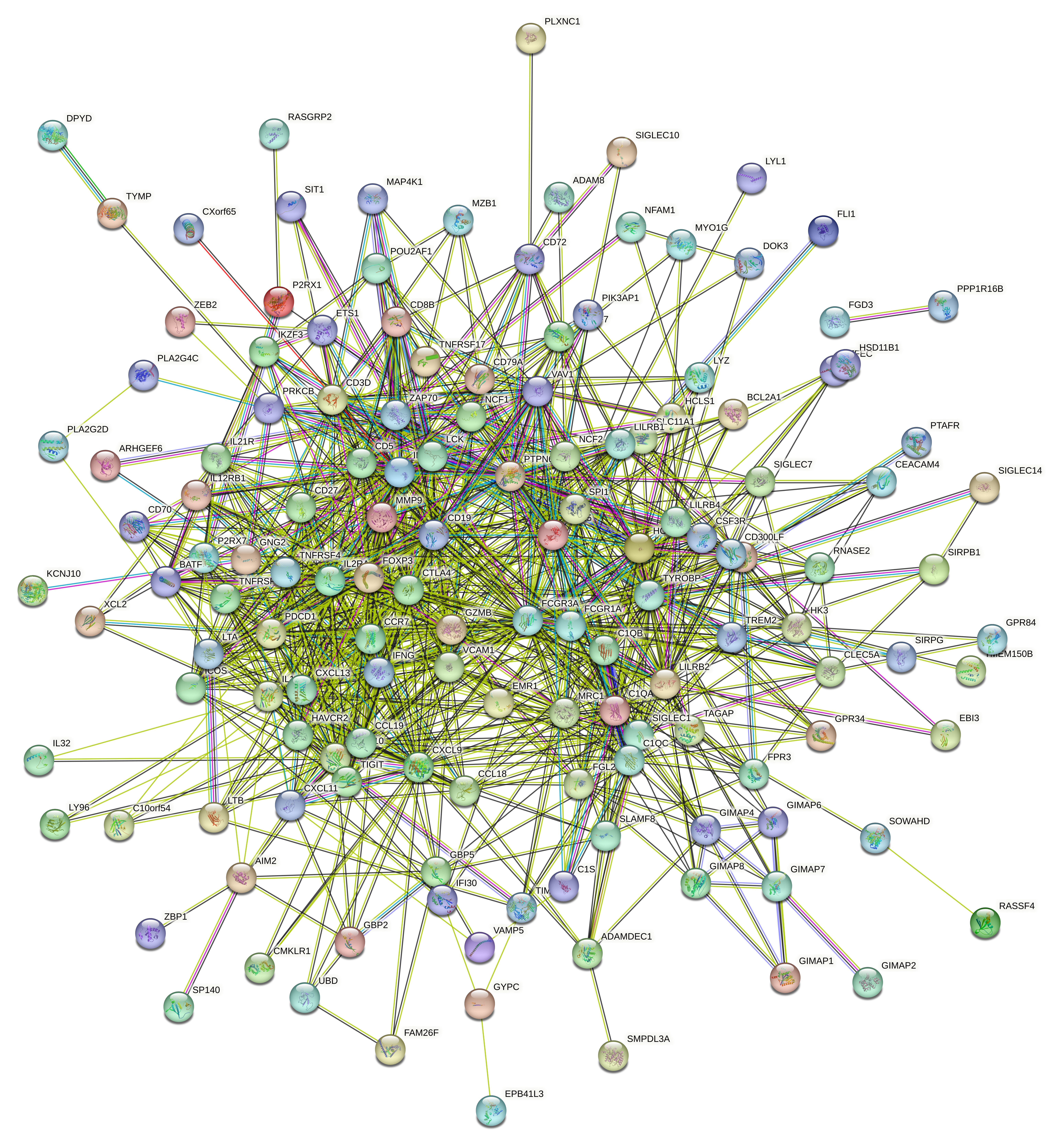

Supplement: Supplementary file 1 [file biomedicines-13-02289-s001.zip › Figure S1.png]

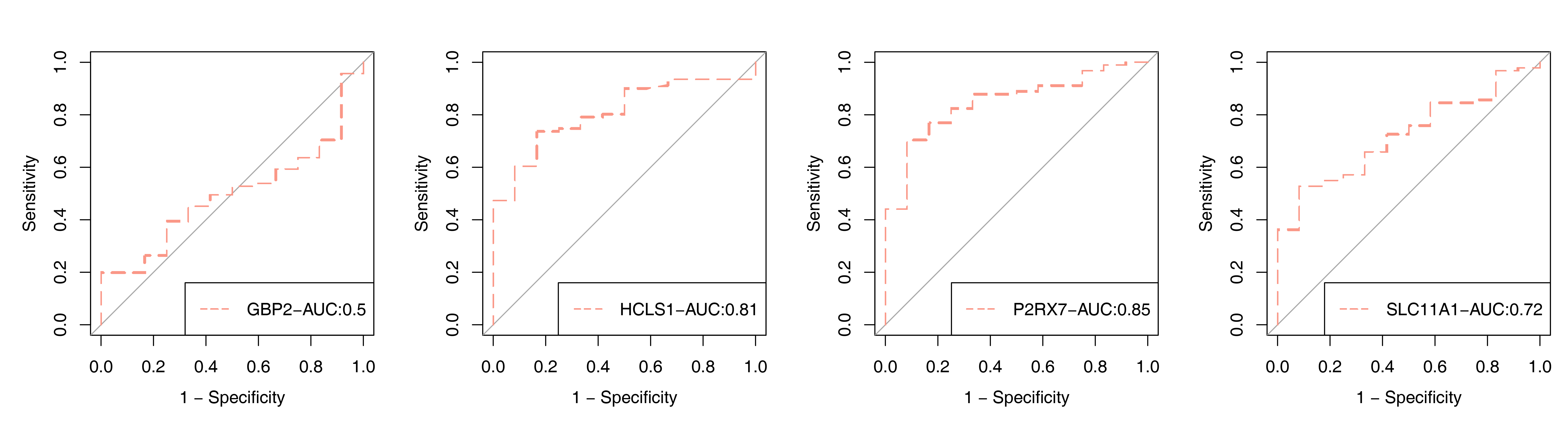

Supplement: Supplementary file 1 [file biomedicines-13-02289-s001.zip › Figure S2.tif]

normal tumor

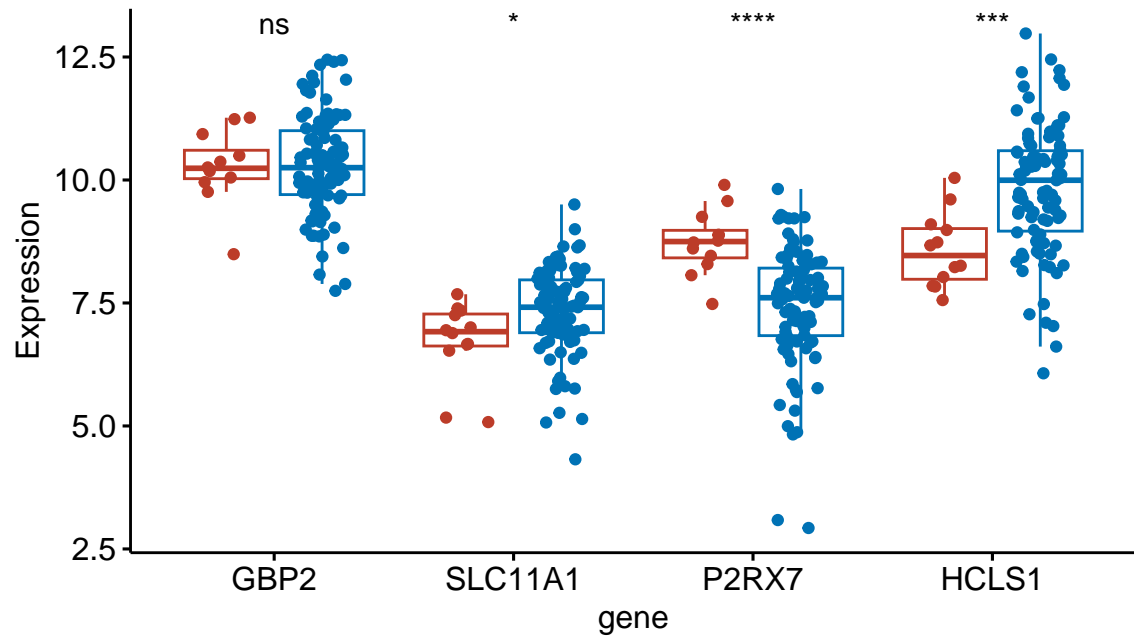

Supplement: Supplementary file 1 [file biomedicines-13-02289-s001.zip › Figure S3.pdf]

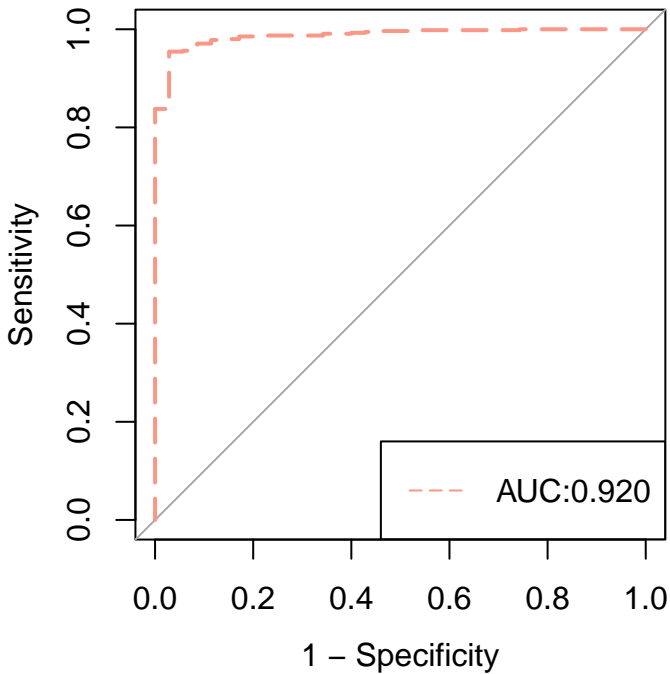

Supplement: Supplementary file 1 [file biomedicines-13-02289-s001.zip › Figure S4.pdf]

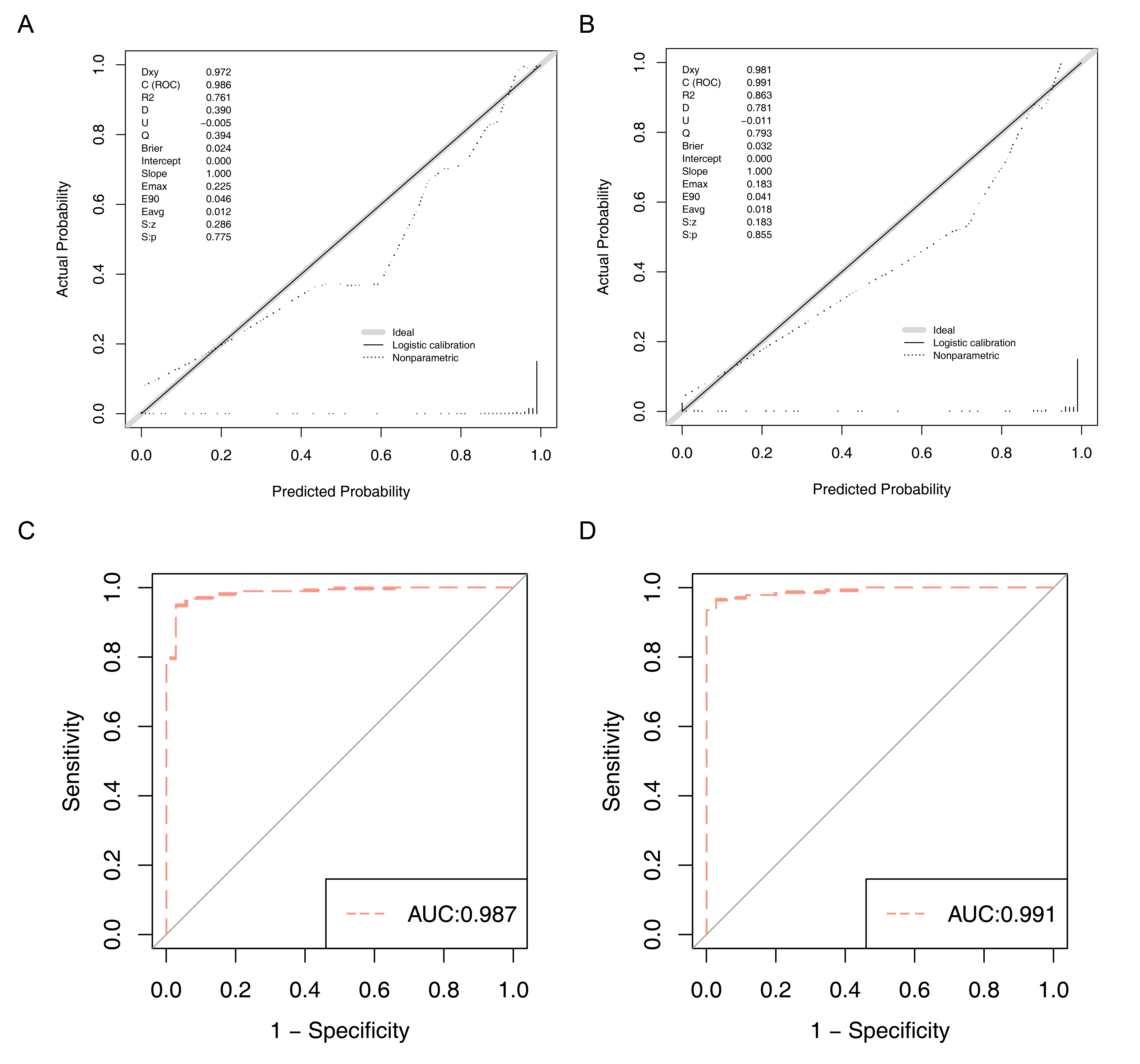

Supplement: Supplementary file 1 [file biomedicines-13-02289-s001.zip › Figure S5.tif]

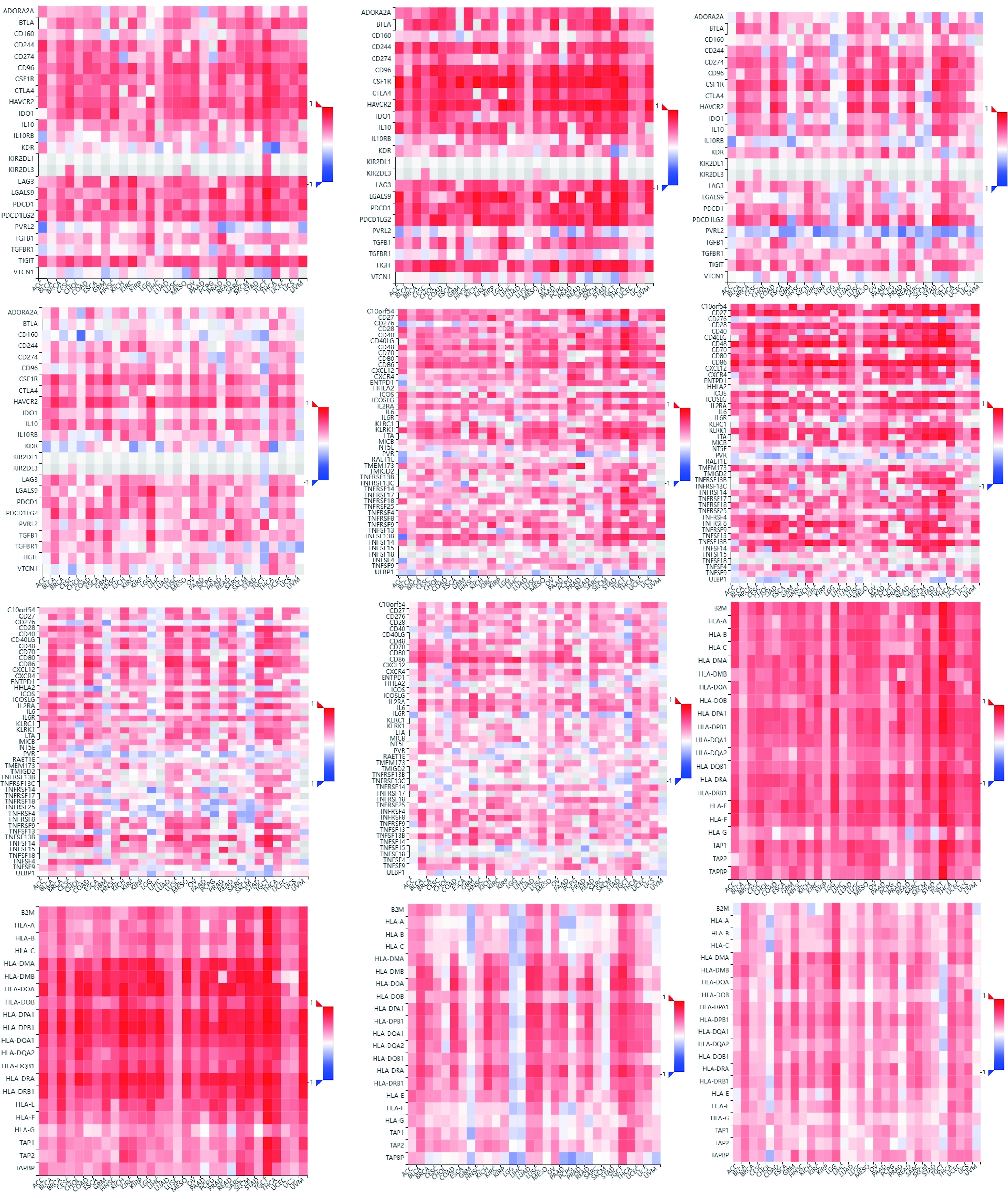

Supplement: Supplementary file 1 [file biomedicines-13-02289-s001.zip › Figure S6.tif]
